# Case report: Significant liver atrophy due to giant cystic pheochromocytoma

**DOI:** 10.3389/fonc.2022.987705

**Published:** 2022-08-30

**Authors:** Qingbo Feng, Hancong Li, Guoteng Qiu, Zhaolun Cai, Jiaxin Li, Yong Zeng, Jiwei Huang

**Affiliations:** ^1^ Department of Liver Surgery and Liver Transplantation Centre, West China Hospital, Sichuan University, Chengdu, China; ^2^ West China School of Medicine, West China Hospital, Sichuan University, Chengdu, China; ^3^ Department of Gastrointestinal Surgery, West China Hospital, Sichuan University, Chengdu, China

**Keywords:** giant cystic pheochromocytoma, liver atrophy, case report, literature review, surgery

## Abstract

**Introduction:**

Pheochromocytoma is a neuroendocrine tumor originating from chromaffin cells in the adrenal medulla. Giant pheochromocytomas with a maximum diameter of over 20 cm are particularly rare.

**Case presentation:**

We present a case of giant cystic pheochromocytoma in a 64-year-old woman who was found to have a right abdominal mass during an ultrasound examination, which is the largest pheochromocytoma ever documented in China. Meanwhile, obvious atrophy of the right lobe of the liver was found in preoperative CT and during the operation. Our literature review identified 20 cases with a diameter of over 20 cm. The average age at diagnosis was 51.7 (range 17–85), and 35% of cases did not exhibit classic symptoms.

**Conclusion:**

Giant pheochromocytoma is an uncommon neoplasm. It can be discovered late due to a lack of clinical manifestations. Diagnosis is dependent on imaging recognition together with catecholamine secretion. Surgical resection is the only curative treatment for such tumors.

## Introduction

Pheochromocytoma is an infrequent catecholamine-secreting neoplasm that arises from chromaffin tissues, which tends to occur in the adrenal medulla (1). The estimated annual incidence of pheochromocytoma is 0.4 to 9.5 per million (2). Hypertension, which can be sustained or paroxysmal, is the most common sign (3). Episodic headache, palpitations, and diaphoresis are the classical triad of clinical features, which can be seen in <25% of patients. At least one component of the triad occurs in slightly less than 50% of patients. These episodes are connected with the catecholamine excess produced by the tumor (4). In addition, local symptoms caused by the giant tumor include stomachache, backache, abdominal distension, and some atypical gastrointestinal symptoms. Commonly, it is a solid tumor and histologically benign (5). Nevertheless, cystic pheochromocytoma is a particular rare entity and is usually asymptomatic but can be fatal due to cardiovascular complications (6). The most accurate metabolic testing for the biochemical diagnosis of this tumor is the elevated plasma-free or 24-h urinary fractionated metanephrines (7). Computed tomography (CT) or magnetic resonance imaging (MRI) is the preference for the initial anatomical localization of the tumor due to its high sensitivity (90%–100%) and reasonable specificity (70%–80%) (4). Once the pheochromocytoma is diagnosed, patients are referred to surgical extirpation, which is the only curative strategy (5). Currently, just a few cases concerning giant cystic pheochromocytomas have been reported worldwide (8). Here, a case of cystic pheochromocytoma with enormous size, a 20 × 15 × 10 cm tumor, is described. The study is reported in agreement with the principles of the CAse REport (CARE) guidelines (9). Additionally, we have performed a literature review on pheochromocytoma measuring 20 cm or greater to update the clinical features of this rare disease. To our best knowledge, this is the largest cystic pheochromocytoma in China as per the available indexed literature.

## Case presentation

On 20 April 2022, a 64-year-old woman was referred to the West China Hospital after an abdominal mass was found on ultrasound examination for mild right upper abdominal discomfort. The patient reported no significant symptoms or past medical history, while physical examination indicated a large mass measuring 20 × 15 cm was palpated at the right upper quadrant. On admission, her blood pressure was 127/89 mmHg, and her heart rate was 78 beats per minute. Initial laboratory investigations concerning biochemistry and hematology examinations were within normal limits.

**Figure 1:**
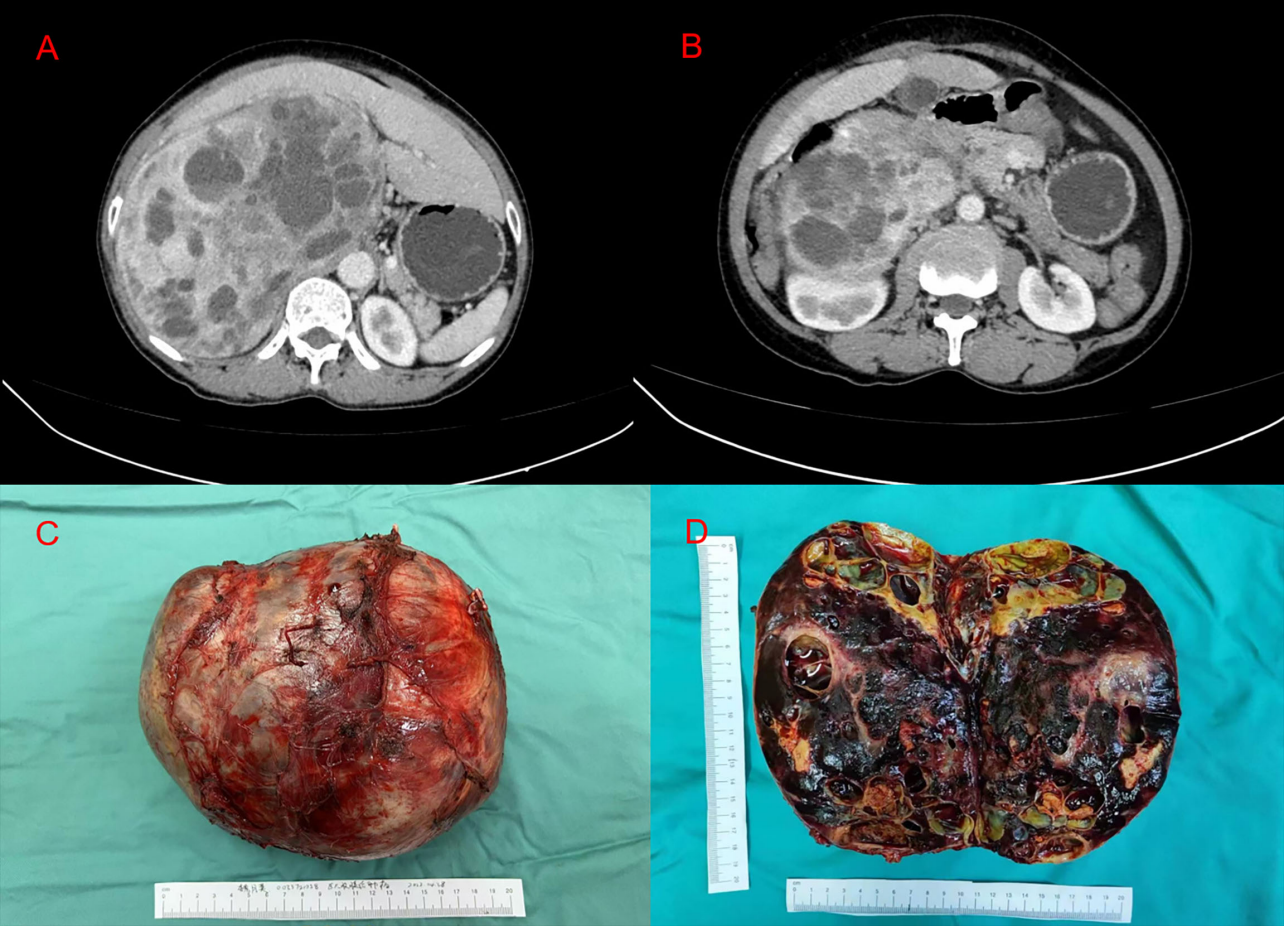
**(A, B)** Abdominal enhanced computed tomography imaging representing the huge tumor between the right lobe of the retroperitoneal liver and the right kidney. **(C)** A macroscopic image of the post-section cystic pheochromocytoma. **(D)** Excised tumor, measuring 20 × 15 × 10 cm.

Abdominal enhanced CT revealed obvious compression atrophy of the liver and a 20 cm × 13 cm mixture of cystic and solid lesions between the right lobe of the retroperitoneal liver and the right kidney (**Figures 1A, B**). Plasma metanephrine and catecholamine measurements were performed, and evidence of pheochromocytoma was increased upon observation of elevated metanephrines and their metabolites in serum (**Table 1**). The patient did not have underlying diseases such as hypertension or diabetes. Before the metabolic testing, she did not take blood pressure medications, acetaminophen, beta- and alpha-adreno receptor blocking drugs, psychotropic medications, or any drugs that might interfere with her metabolism.

**Table 1:** Plasma metanephrines measurements confirming the pheochromocytoma diagnosis.

	Patient values	Units	Ref range	Status
Epinephrine	0.76	nmol/L	<0.34	↑
Norepinephrine	1.89	nmol/L	<5.17	
Dopamine	0.02	nmol/L	<0.31	
Metanephrine	30.72	nmol/L	<0.42	↑
Normetanephrine	96.54	nmol/L	<0.71	↑
3-Methoxytyramine	48.13	pg/ml	<18.40	↑

Based on endocrine consultation, an alpha receptor blocker (phenoxybenzamine) was used for 2 weeks preoperatively. Three days after that, we encouraged the patient to start a salt-rich diet. In addition, an adequate fluid replacement was performed preoperatively. Blood pressure and heart rate of 139/79 mmHg and 77 beats per minute were recorded prior to surgery. The resection of the giant right adrenal pheochromocytoma was completed through a right subcostal incision under general anesthesia (**Figure 1C**). During the operation, the omentum majus widely adhered to the small intestine, abdominal wall, and liver. The right lobe of the liver was squeezed and atrophied by the retroperitoneal tumor, which was located behind the inferior vena cava. Blood pressure fluctuated intraoperatively, between 83 and 157 mmHg for the systolic pressure and between 55 and 92 mmHg for the diastolic pressure. Heart rate was measured between 57 and 87 beats per minute. After surgery, her blood pressure was stable at 112/75 mmHg, and her heart rate was 74 beats per minute. Subsequently, the patient was admitted to an intensive care unit (ICU) for 2 days and then transferred to the ward. Symptomatic supportive treatment was given while the patient was in ICU, including hemodynamic monitoring, oxygen inhalation, analgesia, infection prevention, fluid replacement, and liver function protection. On postoperative day 4, oral feeding was resumed, and the patient was discharged on the eighth day after surgery with no complications. We suggested genetic testing and developed a collaborative, multidisciplinary long-term follow-up plan. With the first outpatient visit 1 month after surgery, an annual follow-up with clinical and biochemical assessment was recommended. The individualized follow-up plan could be flexibly adjusted based on the gene detection and monitoring results.

**Figure 2:**
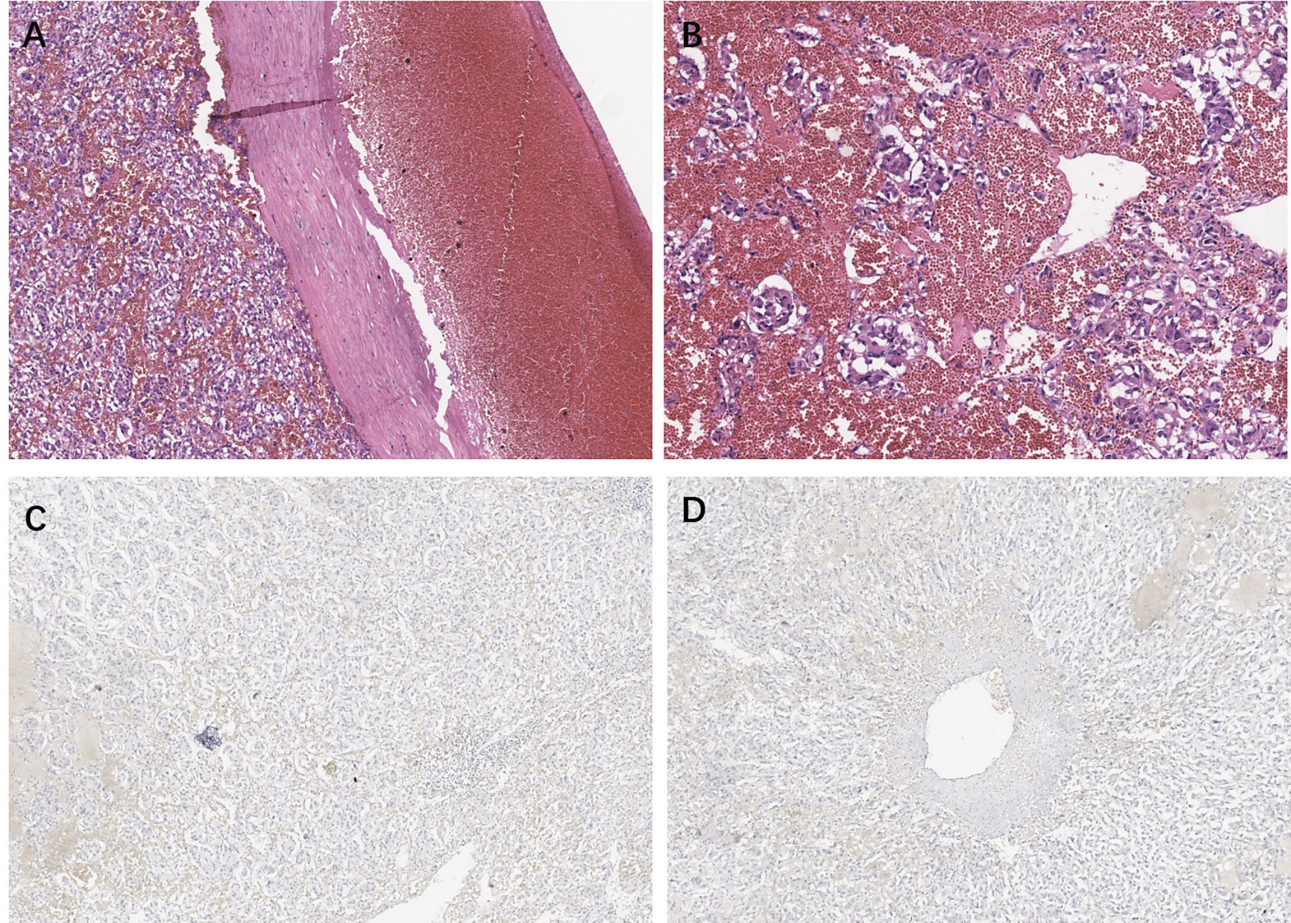
H&E staining in **(A)** ×10 view and **(B)** ×40 view. **(C, D)** CD56 staining immunohistochemistry of the tumor.

Pathological examination of the surgical specimen indicated a necrotic change measuring 20 × 15 × 10 cm and weighing 2,240 g located in the retroperitoneum and right adrenal gland (**Figure 1D**). Hematoxylin–eosin (H&E) staining showed a well-circumscribed tumor surrounded by a fibrous capsule (**Figures 2A, B**). Immunohistochemistry showed marked pleomorphism within a few tumor cells, CD56(+), CgA (+), Syn(+), CK (Pan)(−), MART-1(−), inhibitA(−), S-100(+), INI1(+), Desmin(−), and Myogenin(−) (**Figures 2C, D**). According to the immunohistochemical results, the diagnosis of pheochromocytoma was made. Based on the staging system introduced in the 8th edition of the American Joint Committee on Cancer (AJCC) staging system, the tumor was determined as T2M0N0.

## Discussion

Our case reported a rare cystic pheochromocytoma with a lack of evident endocrine symptoms, resulting in a large size and late diagnosis. The extensive necrosis of the adrenal gland, resulting in reduced production of catecholamines, and the retention of these hormones in the capsule mass after secretion may be the explanation for the absence of symptoms. As a result, the time to diagnosis was delayed, and the tumor size was larger once it was detected.

Since serious complications such as myocardial infarction, arrhythmias, dissection aortic aneurysms, heart failure, malignant hypertension, and sudden death can be caused by pheochromocytoma, it might give rise to premature mortality if overlooked (10, 11). In addition to this, cases of misdiagnosis of cystic pheochromocytoma such as pancreatic cystic tumor (12, 13), hepatic cystic tumor (14), simple adrenal cyst (15), and liver abscess (16) under CT scan have also been previously documented. Hence, it is essential to investigate the characteristics of this tumor.

To date, a total of 20 cases (5, 8, 17–33) of giant pheochromocytoma ≥20 cm were documented since the tumor was first described in 1886 by Fränke (34). We reviewed previous reports and summarized the clinical characteristics (**Table 2**). The average age at the discovery of giant pheochromocytoma was 51 (range 17–85) years. No significant difference was observed in gender, with slightly more women (11 cases). Additionally, similar findings were revealed in tumor histopathology (five and six cases manifested as malignant and benign, respectively). More cases had tumors that occurred on the left (11 cases) side of the abdomen than on the right (seven cases), and the rest of the articles did not explicitly state the site. Most clinical manifestations were asymptomatic (seven cases) and were often discovered occasionally during a physical examination. In addition, backache (three cases), abdominal pain (two cases), and chest pain (two cases) are relatively common chief complaints. What is more, non-endocrine manifestations, such as abdominal swelling, nausea, constipation, fatigue, and weight loss, could also be presented as the patient’s chief complaints.

**Table 2:** A summary of reported giant pheochromocytomas with a maximal diameter greater than 20 cm, arranged by the largest to smallest maximum diameter.

	First author	Year	Country	Size (cm)	Weight(g)	Sex	Age	Location	Histopathologicalevaluation	Operation approach	Component	Chief complaint
1	Grissom	1979	USA	45 × 25	3,000+	F	54	Left abdomen	NA	Open	NA	Asymptomatic
2	Arikan	2021	Turkey	30 × 23	NA	M	54	Right abdomen	Benign	Open	Cystic	Hypertension
3	Costa	2008	Brazil	30	NA	M	46	Right abdomen	Malignant	Open	Cystic	Abdominal pain
4	Basso	1996	Italy	29 × 21 × 12	4,050	M	47	Left abdomen	Malignant	Open	Solid	Asymptomatic
5	Karumanchery	2012	England	28 × 16 × 13	2,300	F	85	Left abdomen	NA	Open	Cystic	Back pain
6	Maharaj	2017	Trinidad and Tobago	27 × 18 × 12	3,315	F	50	Left abdomen	Low risk of malignancy	Open	NA	Back pain, fatigue, and weight loss
7	Samejima	2019	Japan	27	NA	M	45	Right abdomen	Benign	Open	Cystic	Abdominal swelling
8	Gupta	2016	India	25 × 17 × 15	2,750	F	65	Left abdomen	Benign	Open	Cystic	Left upper abdominal lump
9	Okada	2016	Japan	24 × 23 × 16	5,900	F	43	Abdomen	NA	Open	NA	Vulva edema
10	Díaz-Roldán	2021	Spain	23 × 15 × 10	NA	F	57	Right abdomen	Benign	Open	NA	Morning headache
11	Suga	2000	Japan	21 × 13 × 21	3,900	M	48	Left abdomen	NA	Open	Cystic	Asymptomatic
12	Terk	1993	USA	21 × 20 × 11	2,870	M	35	Abdominal and pelvic cavity	NA	Open	NA	Constipation
13	Arcos	2009	Canada	21 × 17 × 11	NA	F	36	Left abdomen	Malignant	Open	NA	Lower back pain
14	Soufi	2012	India	21 × 15	NA	F	17	Right abdomen	Malignant	Open	Cystic	Asymptomatic
15	Cajipe	2017	USA	21 × 12 × 10.5	1,773	F	56	Left abdomen	Benign	Open	Cystic	Nausea, vomiting, fainting spells
16	Ologun	2017	USA	20.5 × 18 × 10	2,582	F	55	Right abdomen	NA	Open	NA	Abdominal pain, chest pain, palpitation,
17	Melegh	2002	Hungary	20	NA	M	55	Left abdomen	NA	Open	Cystic	Asymptomatic
18	Korgali	2014	Turkey	20 × 17 × 9	1,736	M	63	Left abdomen	Malignant	Open	Solid-cystic	Chest pain, sweating, nausea
19	Current case	2022	China	20 × 15 × 10	2,240	F	64	Right abdomen	Benign	Open	Cystic	Asymptomatic
20	Jiang	2017	China	20 × 14 × 5	NA	M	45	Left abdomen	NA	Open	NA	Asymptomatic

Till now, the largest pheochromocytoma in the world was recorded to be 45 × 20 cm originally by Grissom et al. in 1979 (17). The current case turned out to be the largest cystic pheochromocytoma in China.

One of the unusual features of the present case is the cystic components. Most of the time, pheochromocytoma is a solid neoplasm originating from the adrenal medulla (29, 35), while cystic pheochromocytoma is a rare neuroendocrine tumor, with only a few described in former studies (5, 18, 19, 21, 22, 25, 29, 31). Goldberg et al. and Samejimaonce et al. successively found that the cystic fluid contains high concentrations of catecholamines and metanephrines (22, 35). Extensive hemorrhage followed by necrosis with cyst formation and subsequent resorption of the contents may be the postulated mechanism for this cystic degeneration in the tumor. Furtherly, extensive hemorrhage was likely triggered by the tumor outgrowing its vascular supply (36). This cystic change has also been demonstrated in other primary adrenal neoplasms, either benign cortical adenomas and hemangioma or primary and metastatic malignant adrenal lesions (37).

Notably, despite the surgical margin being histologically negative, the nature of the cystic pheochromocytoma should be interpreted with caution.

As with many other neuroendocrine tumors, it is almost impossible to confirm whether a tumor is benign or malignant only by histological criteria (5, 38). According to 2017 WHO classification of endocrine tumors, all pheochromocytomas are deemed to have metastatic potential, replacing the previous term “malignant” (39). This approach is maintained in the 2022 WHO classification (40). Thus, many researchers are attempting to develop scoring or classification systems that would predict the future behavior of pheochromocytoma. Multifactorial assessment, including tumor size (≥5 cm), Ki-67 index, SDHB mutation, and the dopaminergic phenotype have been suggested to assess the metastatic potential (40–42). These methods for screening for SDHx mutations are reasonable for rapid identification of patients at high risk of metastasis. However, accurate genetic testing remains essential (40). In addition to this, the Pheochromocytoma of the Adrenal gland Scaled Score (PASS) system has carried out a meaningful attempt (43). This is a risk-stratification system entirely based on histological features. However, the PASS score seems to be more reliable concerning the negative predictive value for the absence of metastatic behavior (44). The Grading system for Adrenal Pheochromocytoma and Para-ganglioma (GAPP) system, based on the growth pattern, cellularity, comedo-type necrosis, and vascular or capsular invasion, complemented by the Ki-67 index and catecholamine type demonstrates good predictive performance (45).

However, the problem concerning the label “poorly differentiated” for these neoplasms that are not poorly differentiated in the context used for high-grade neuroendocrine tumors has been noted by the WHO/International Agency for Research on Cancer (IARC) classification. Currently, COmposite Pheochromocytoma/para-ganglioma (COPPS) Prognostic Score classification systems, with a sensitivity of 100% and specificity of 95%, are proposed. This score focuses on clinicopathological criteria, including tumor size (>7 cm), necrosis, vascular invasion, and SDHB immunohistochemical staining. As the study was only recently published, it has not been independently verified (40). At present, the latest version of WHO classification does not recognize any of these systems but at the same time does not discourage their use in individual practices (40). Thereby, subsequent follow-up and metastasis monitoring are still critical to patient care.

Upon suspicion of pheochromocytoma, verification can be performed by biochemical testing (46). Among hormonal assay tests, elevated levels of metanephrines in 24-h urine or plasma are of the highest suggestibility (47). Anatomical imaging should be followed as the first modality if biochemical tests indicate the presence of pheochromocytomas. Abdominal or pelvic CT scans are helpful and highly recommended (47, 48). Other imaging studies, such as abdominal/pelvic multiphasic CT or MRI scans or nuclear medicine imaging, including meta-iodobenzylguanidine (MIBG) scintigraphy, FDG-PET/CT scans, and DOTA-SSA PET/CT scans, should be performed as appropriate if metastatic or multifocal disease is suspected (47). Functional imaging enables accurate diagnosis of tumor recurrence or metastasis that might go undetected by anatomical imaging (49). Regarding differential diagnosis, [^18^F]-FDG can be helpful to determine whether the tumors were benign or indicate primary malignant adrenal diseases in non-functioning adrenal masses with inconclusive CT/MRI imaging (50). Further, the latest review recommends specific nuclear imaging for the different clusters of pheochromocytoma when conducting personalized surveillance and management (39, 50). Based on genetic testing, [^68^Ga]-DOTA-SSA PET/CT is demonstrated as the most sensitive functional imaging modality for cluster 1A, while [^18^F] FDOPA PET/CT is more sensitive for cluster 1B and cluster 2 tumors (39). Our CT image presents a sporadic isolated adrenal mass, and pheochromocytoma was highly suspected based on the imaging features. In addition, given the patient’s financial situation and her wishes, we finally had no nuclear medical examination performed.

Once a pheochromocytoma is diagnosed, surgery is the preferred treatment whenever possible, regardless of its nature, component, and size. In addition to the conventional open approach, laparoscopic surgery has emerged as a favorable approach due to its ability to decrease hospitalizations, transfusion, and analgesia requirements. However, considering tumor size and location, the open operation is still recommended when tumors are with malignant potential, bilateral, and larger than 8 cm. Currently, there are four reports of laparoscopic excision of pheochromocytoma larger than 10 cm, and the maximum is 14 cm (51–54). If metastatic disease is present, primary tumor removal/debulking surgery may also be performed to alleviate symptoms and signs of catecholamine overdose or local symptoms.

Intraoperative hemodynamic monitoring, especially management of the hypertensive crisis by an experienced anesthesiologist, is essential for optimal surgical outcomes. Arrhythmias are common during the procedure, and intravenous administration of esmolol and lidocaine are effective measures. After removal of the origin of excess catecholamines in the circulation, postoperative hypotension may be intractable and is usually managed with intravenous fluid replacement (sometimes with vasopressors). Intravenous glucose prevents hypoglycemia, which occurs in 10%–15% of patients due to the elimination of the inhibitory effect of catecholamines on insulin secretion.

Postoperatively, although there has been no accurate consensus on follow-up, long-term multidisciplinary follow-up is indispensable. Since genotype–phenotype presentations have been proved to be associated with pheochromocytoma personalized management, gene detection is encouraged for patients. So far, 14 different susceptibility genes of pheochromocytomas have been documented: NF1, RET, VHL, SDHD, SDHC, SDHB, EGLN1/PHD2, KIF1β, SDH5/SDHAF2, IDH1, TMEM127, SDHA, MAX, and HIF2α (55). Among them, mutations of SDHB lead to tumor metastasis in 40% or more of affected patients (56). The 5th edition of the WHO Classification encourages routine use of SDHB immunohistochemistry (40). Vigilant imaging examinations should be performed as required.

Collectively, we reported a fairly rare case of a giant cystic pheochromocytoma and provided an up-to-date literature review of patients with such tumors larger than 20 cm. Large pheochromocytomas are usually asymptomatic and require individualized surgery. The prognosis is generally well, but long-term follow-up is required to monitor the tumor for metastasis or recurrence.

## Data availability statement

The original contributions presented in the study are included in the article/supplementary material. Further inquiries can be directed to the corresponding author.

## Ethics statement

Written informed consent was obtained from the individual for the publication of any potentially identifiable images or data included in this article.

## Author contributions

QF and HL drafted and revised the manuscript. GQ and ZC collected the data and revised the manuscript. JL revised the manuscript for content. YZ and JH designed the study and revised the manuscript. All authors contributed to the article and approved the submitted version.

## Funding

This work was supported by grants from the National Key Technologies R&D Program (2018YFC1106800), the Natural Science Foundation of China (82170621, 82070644, 81800564, and 81770615), and the 1.3.5 project for disciplines of excellence, West China Hospital, Sichuan University (ZYJC18008).

## Conflict of interest

The authors declare that the research was conducted in the absence of any commercial or financial relationships that could be construed as a potential conflict of interest.

## Publisher’s note

All claims expressed in this article are solely those of the authors and do not necessarily represent those of their affiliated organizations, or those of the publisher, the editors and the reviewers. Any product that may be evaluated in this article, or claim that may be made by its manufacturer, is not guaranteed or endorsed by the publisher.
